# Functional analyses of miRNA-146b-5p during myogenic proliferation and differentiation in chicken myoblasts

**DOI:** 10.1186/s12860-020-00284-z

**Published:** 2020-05-29

**Authors:** Jeong Hyo Lee, Seo Woo Kim, Ji Seon Han, Seung Pyo Shin, Sang In Lee, Tae Sub Park

**Affiliations:** 1 Institute of Green-Bio Science and Technology, Seoul National University, Pyeongchang-gun, Gangwon-do 25354 South Korea; 2 Graduate School of International Agricultural Technology, Seoul National University, Pyeongchang-gun, Gangwon-do 25354 South Korea; 3 Department of Animal Biotechnology, Kyungpook National University, Sangju, Gyeongsangbuk-do 37224 South Korea

## Abstract

**Background:**

In the poultry and livestock industries, precise genetic information is crucial for improving economic traits. Thus, functional genomic studies help to generate faster, healthier, and more efficient animal production. Chicken myoblast cells, which are required for muscle development and regeneration, are particularly important because chicken growth is closely related to muscle mass.

**Results:**

In this study, we induced expression of microRNA-146b-5p mediated by the *piggyBac* transposon system in primary chicken myoblast (pCM) cells. Subsequently, we analyzed and compared the proliferation and differentiation capacity and also examined the expression of related genes in regular pCM (rpCM) cells and pCM cells overexpressing miRNA-146b-5p (pCM-146b OE cells). pCM-146b OE cells showed increased proliferation and upregulated gene expression related to cell proliferation. In addition, next-generation sequencing analyses were performed to compare global gene expression patterns between rpCM cells and pCM-146b OE cells. We found that the higher proliferation in pCM-146b OE cells was the result of upregulation of gene sets related to the cell cycle. Moreover, miRNA-146b-5p overexpression had inhibitory effects on myotube differentiation in pCM cells.

**Conclusions:**

Collectively these results demonstrate that miR-146b-5p is closely related to the proliferation and differentiation of chicken myogenic cells as a modulator of post-transcription.

## Background

Since the genome sequences of avian species have become available, research has aimed to increase muscle mass, enhance muscle regeneration, and reduce fatty acid accumulation to improve growth. Understanding the genes or genetic markers involved in biological functions and regulatory pathways can help to improve economically important traits in the poultry industry [1–3]. Thus, functional genomic studies are powerful and effective for investigating the modulatory mechanisms between cell proliferation and differentiation, in particular in skeletal muscles [4–7]. Our study was conducted in chicken myoblasts derived from embryonic tissue. Myoblasts are derived from satellite cells, which are a precursor to myogenesis [8]. In the quiescent satellite cell stage, PAX7, a critical marker of undifferentiated myoblasts, is highly expressed. After activation, myoblasts start to proliferate, decrease expression of PAX7, and increase expression of MYOD, a myogenic regulatory factor (MRF). Then they enter the stage of terminal differentiation. In this stage, expression of MYOD decreases, and expression of markers of terminal differentiation such as Myogenin and Desmin increases. Eventually myoblast cells form new myotubes, which in turn form new myofibers [9]. Therefore, myoblasts are closely related to muscle growth, which is an economically important trait of domestic animals.

MicroRNA (miRNA) is a small non-coding RNA molecule that can regulate targeted gene expression by specific mRNA degradation and translational inhibition [10–12]. There are numerous reports of miRNAs controlling developmental and cellular processes such as cell proliferation, differentiation, and tissue specification [13, 14]. In addition, some miRNAs, such as miRNA-1 and miRNA-206, control myogenesis in mammals [15, 16]. miRNA-146b (miR-146b) is well conserved in most vertebrates and has many biological functions in innate immunity, inflammation, and cell senescence [17–19]. Dicing the pre-miR-146b stem-loop creates two different miRNA species: miR-146b-5p as a major form and miR-146b-3p as a minor form [17–19]. miR-146b-5p is a key regulator of muscle regeneration and myoblast differentiation in mice [20]. Furthermore, miR-146b-3p controls myoblast proliferation and differentiation in chicken [21].

However, additional research is required because it is not clear how miR-146b-5p affects myogenic differentiation, and there are no reports addressing its effects on chicken myogenesis. Thus, in this study, we designed and constructed a miRNA expression vector system to overexpress miR-146b-5p in pCM cells using the *piggyBac* transposon system, which previous studies have demonstrated to be an efficient transgene delivery system [22, 23]. miR-146b-5p increased proliferation and decreased differentiation by regulating genes related to the cell cycle in chicken myoblasts.

## Results

### miR-146b-5p overexpression in pCM cells

**Fig. 1:**
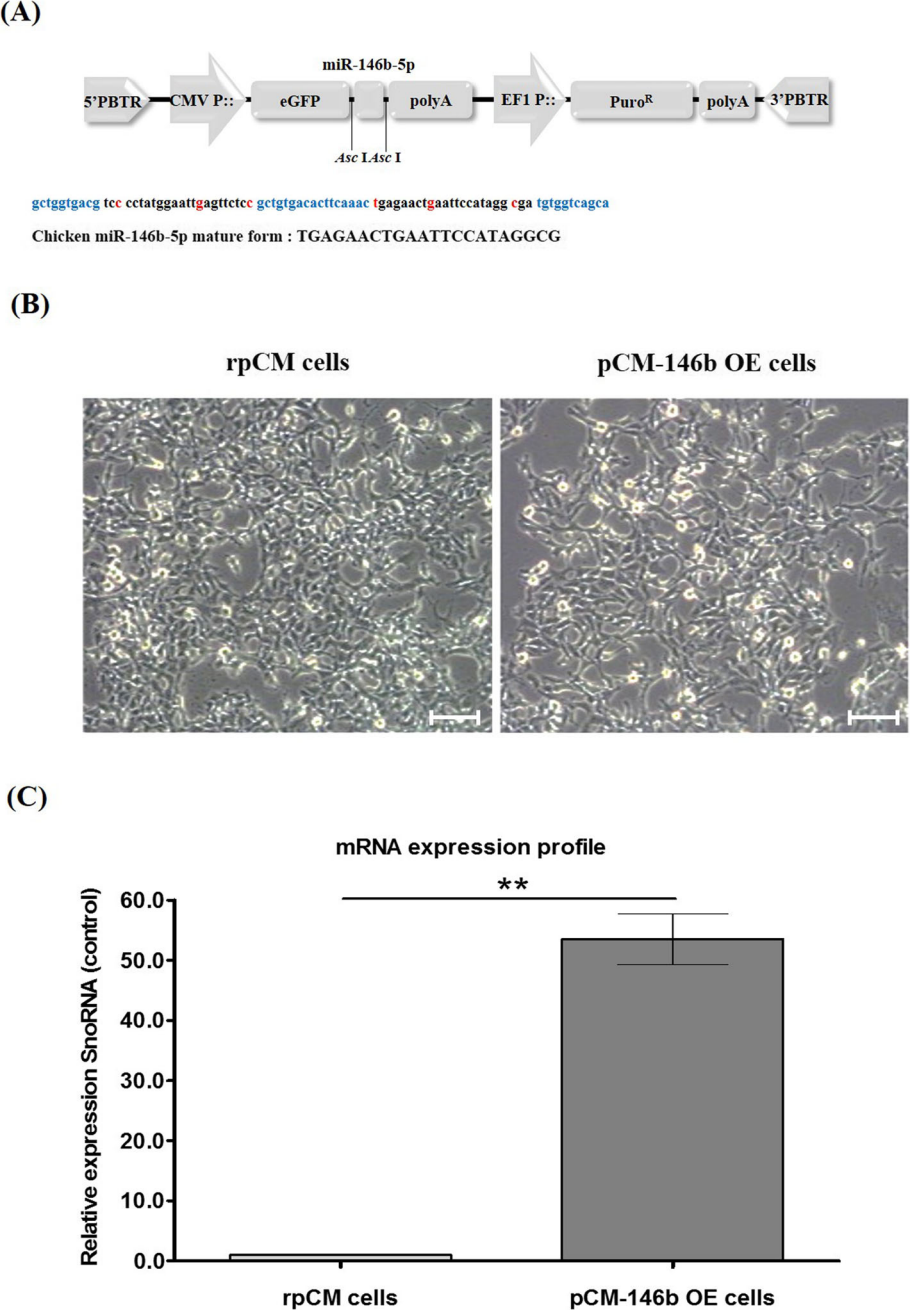
Design of the chicken miRNA-146b-5p expression vector and characterization of miR-146b-5p overexpression in primary chicken myoblast (pCM) cells. **a** The expression vector of piggyBac CMV-GFP-mir146b-5p. The cytomegalovirus and EF1 promoter controlled the expression of GFP-miR146b-5p and the puromycin resistance gene, respectively. **b** Morphology of regular pCM (rpCM) and pCM cells overexpressing miR-146b-5p (pCM-146b OE cells; scale bar = 100 μm). **c** mRNA expression profiles of miR-146b-5p were compared between regular pCM and pCM-146b OE cells by qRT-PCR (**p < 0.01)

Based on our previous study using a miRNA expression system [24], we designed and constructed a *piggyBac* transposon-mediated miR-146b-5p overexpression vector (*piggyBac* CMV-GFP-miRNA-146b-5p; Fig. 1a). Two copies of miR-146b-5p were simultaneously transcribed with the GFP transgene under the CMV promoter (Fig. 1a). This miRNA expression cassette system was used to overexpress the targeted miRNA and also to visualize GFP in the transfected cells. The rpCM and pCM-146b OE cells showed no differences in terms of morphological features (Fig. 1b). Quantitative RT-PCR (qRT-PCR) was performed to determine the overexpression of miR-146b-5p in pCM-146b OE cells. Expression of miR-146b-5p was significantly upregulated in pCM-146b OE cells compared to rpCM cells (Fig. 1c).

### Characterization of pCM-146b OE cells in an undifferentiated state

**Fig. 2:**
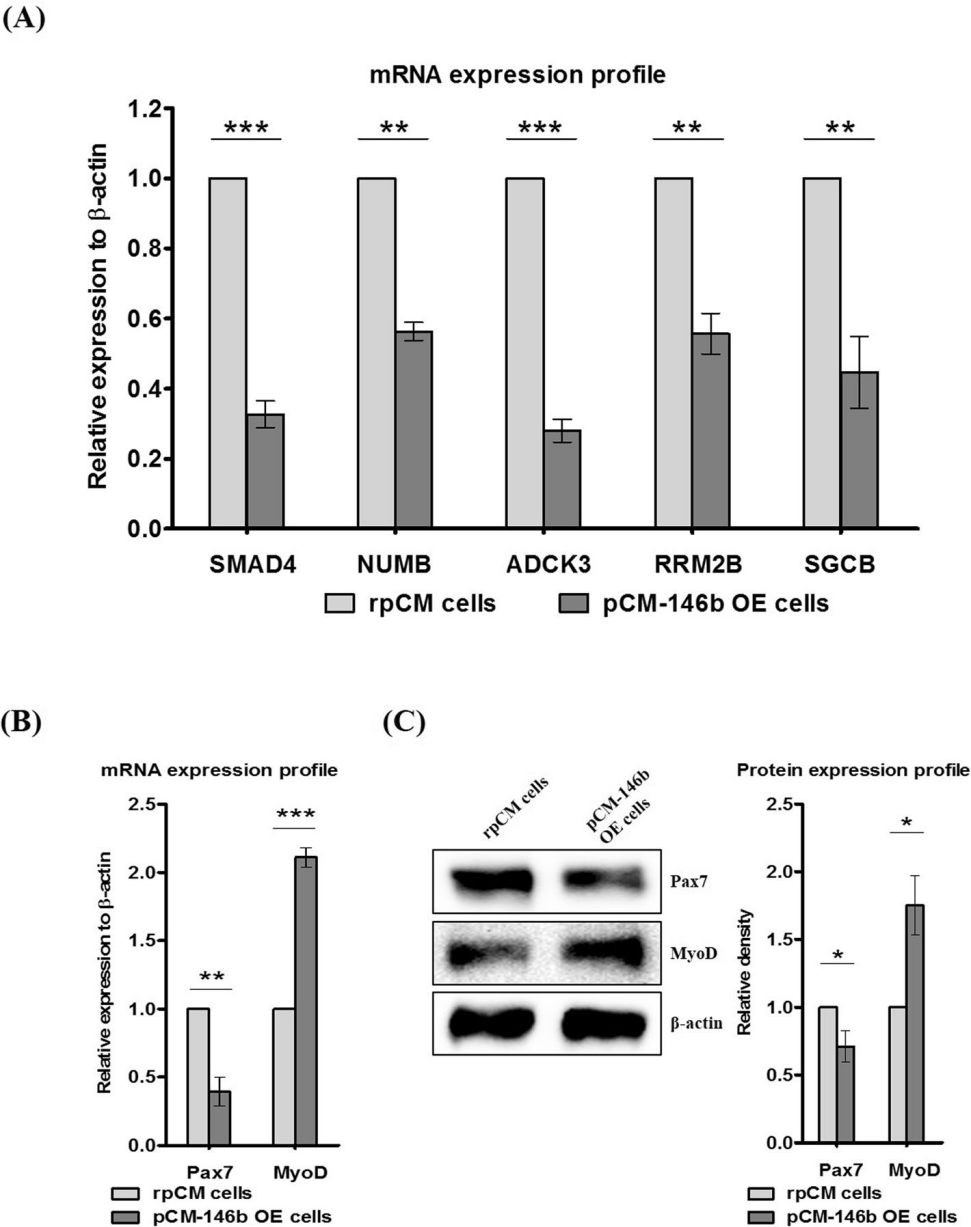
Gene expression analyses of undifferentiated primary chicken myoblast (pCM) cells by quantitative RT-PCR (qRT-PCR) and Western blotting. **a** mRNA expression profiles of target transcripts of miR-146b-5p (*Smad4*, *Numb*, *Asck3*, *Rrm2b*, and *Sgcb*) were compared between regular pCM (rpCM) and pCM cells overexpressing miRNA-146b-5p (pCM-146b OE cells) by qRT-PCR (*n* = 3; **p < 0.01, ***p < 0.001). **b** mRNA expression profiles of *Pax7* and *MyoD* were compared between rpCM and pCM-146b OE cells by qRT-PCR (*n* = 3; **p < 0.01, ***p < 0.001). **c** Protein expression profiles of PAX7 and MYOD were compared between undifferentiated regular pCM and pCM-146b OE cells. The graph represents a density comparison of Western blotting results (*n* = 3; *p < 0.05)

To examine gene expression patterns of the myogenic markers and targets of miR-146b-5p in the undifferentiated state, we compared results of qRT-PCR and Western blotting between rpCM and pCM-146b OE cells. Based on miRBase (http://www.mirbase.org), we selected predicted targets of miR-146b-5p and analyzed their expression patterns in pCM-146b OE cells. All predicted target transcripts (*Smad4*, *Numb*, *Asck3*, *Rrm2b*, and *Sgcb*) were significantly downregulated in pCM-146b OE cells compared to rpCM cells (Fig. 2a). Expression of *Pax7*, a critical marker of undifferentiated myoblasts, was downregulated, whereas expression of the MRF *MyoD* was upregulated (Fig. 2b). Western blotting confirmed the downregulated and upregulated expression of PAX7 and MYOD, respectively (Fig. 2c). These results indicate that miR-146b-5p may be involved in transcriptional regulation of myogenic genes in pCM cells.

### Overexpression of miR-146b improves the proliferation of pCM cells

**Fig. 3:**
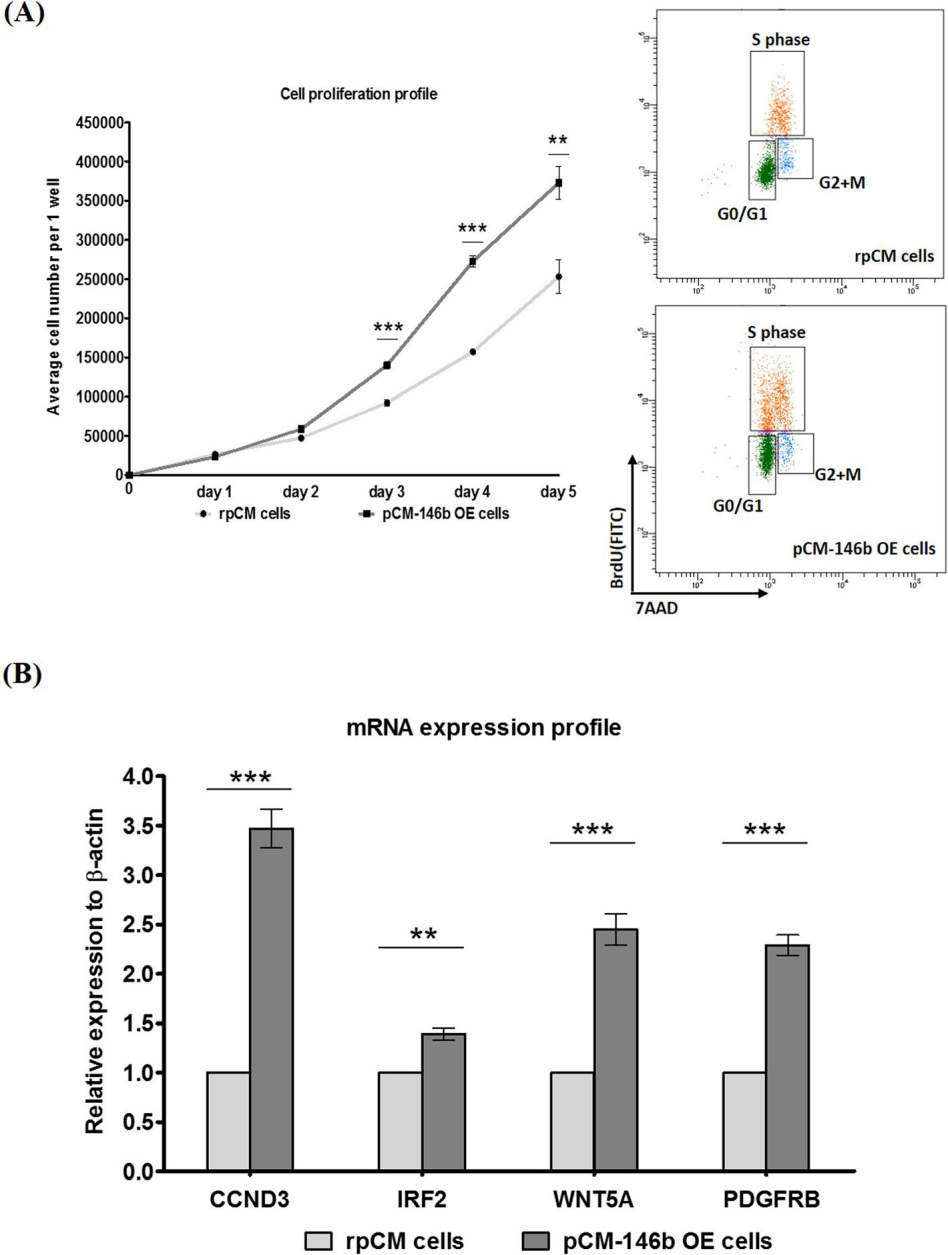
Proliferation and gene expression analyses during primary chicken myoblast (pCM) cell growth. **a** Cell proliferation was compared between GFP-expressing pCM (pCM-GFP cells) and pCM cells overexpressing miRNA-146b-5p (pCM-146b OE cells) over 5 days (*n* = 3; **p < 0.01, ***p < 0.001). The right panels show flow cytometry analyses with BrdU for cell cycles, indicating that subsets of pCM-146b OE cells in S phase were significantly increased compared to rpCM cells (rpCM cells: 22.6 ± 0.9% vs. pCM-146b OE cells: 47.8 ± 6.0%, *n* = 3). **b** mRNA expression profiles of *Ccnd3*, *Irf2*, *Wnt5a*, and *Pdgfrb* were compared between regular pCM and pCM-146b OE cells by qRT-PCR (*n* = 3; **p < 0.01, ***p < 0.001)

It is intriguing that pCM-146b OE cells had a higher proliferative growth rate. To exclude the influence of GFP, which was inserted into the miR-146b-5p overexpression vectors, we compared proliferation in pCM-GFP cells and pCM-146b OE cells. pCM-146b OE cells had a higher growth rate than pCM-GFP cells after 3 days of in vitro culture (Fig. 3a). In addition, cell cycle analyses with BrdU showed that subsets of pCM-146b OE cells in S phase were significantly increased compared to rpCM cells (rpCM cells: 22.6 ± 0.9% vs. pCM-146b OE cells: 47.8 ± 6.0%, *n* = 3; Fig. 3a). Furthermore, we analyzed genes related to cell proliferation (*Ccnd3*, *Irf2*, *Wnt5a*, and *Pdgfrb*) by qRT-PCR. All transcripts, which are positive regulators of proliferation, were upregulated in pCM-146b OE cells (Fig. 3b). These results suggest that miR-146b-5p has effects on the proliferation of skeletal muscle in chicken myoblast cells.

### Global gene expression analyses by RNA sequencing in pCM-146b OE cells

**Fig. 4:**
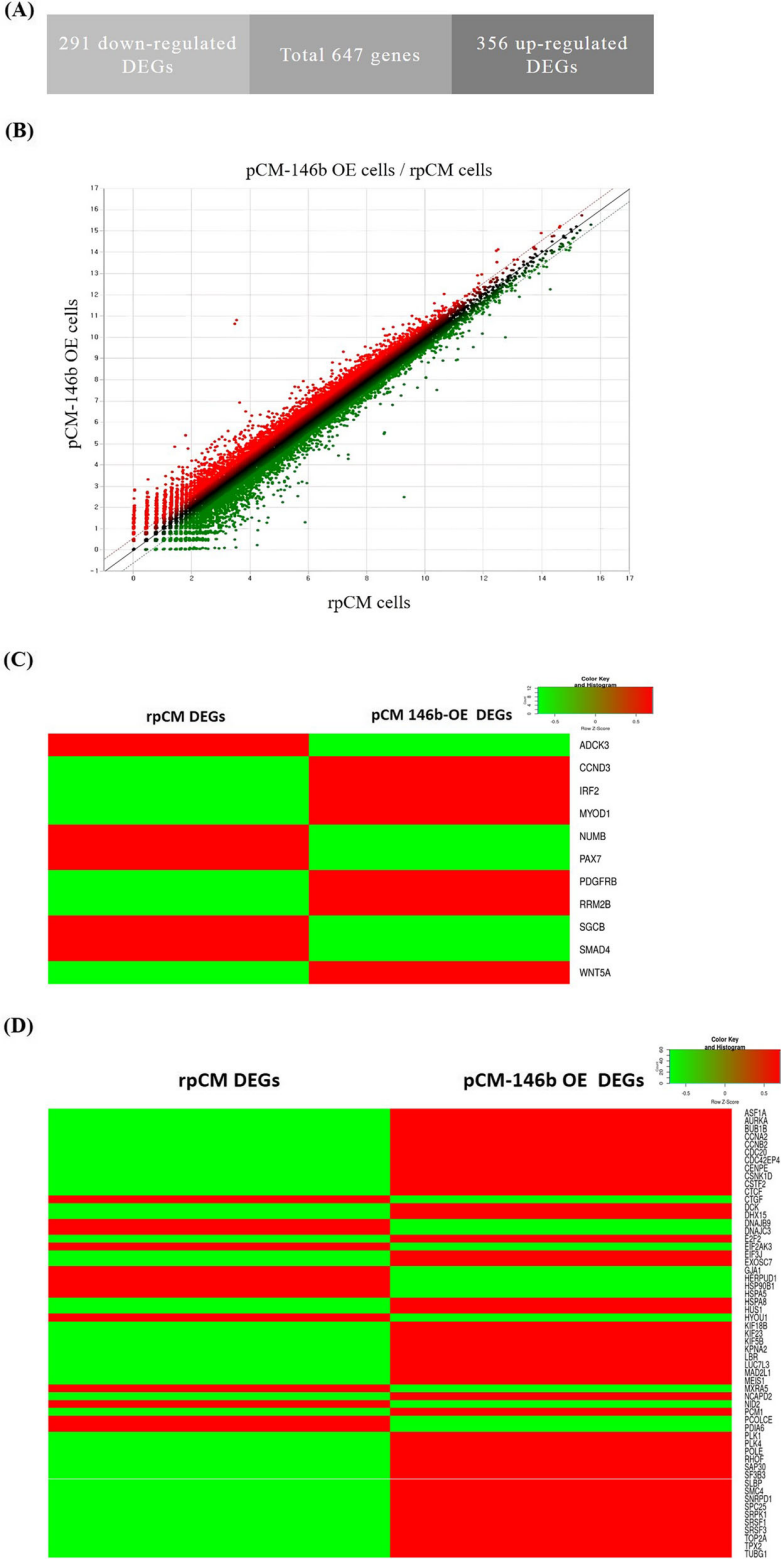
mRNA sequencing analyses of regular primary chicken myoblast (rpCM) cells and pCM cells overexpressing miRNA-146b-5p (pCM-146b OE cells). **a** Differentially expressed genes (DEGs) between the rpCM cells and pCM-146b OE cells were identified with a *p* cutoff of 0.001 and a fold change cutoff of 1.5. **b** Scatter plot analyses of DEGs. The red and green dots indicate the expression of upregulated and downregulated genes in the pCM-146b OE cells, respectively. **c** Heatmap analyses for mRNA sequencing data visualizing the different expression patterns of the predicted target transcripts and genes related to the cell cycle. **d** The gene sets globally up- and downregulated between rpCM and pCM-146b OE cells were visualized by heatmap analyses

**Fig. 5:**
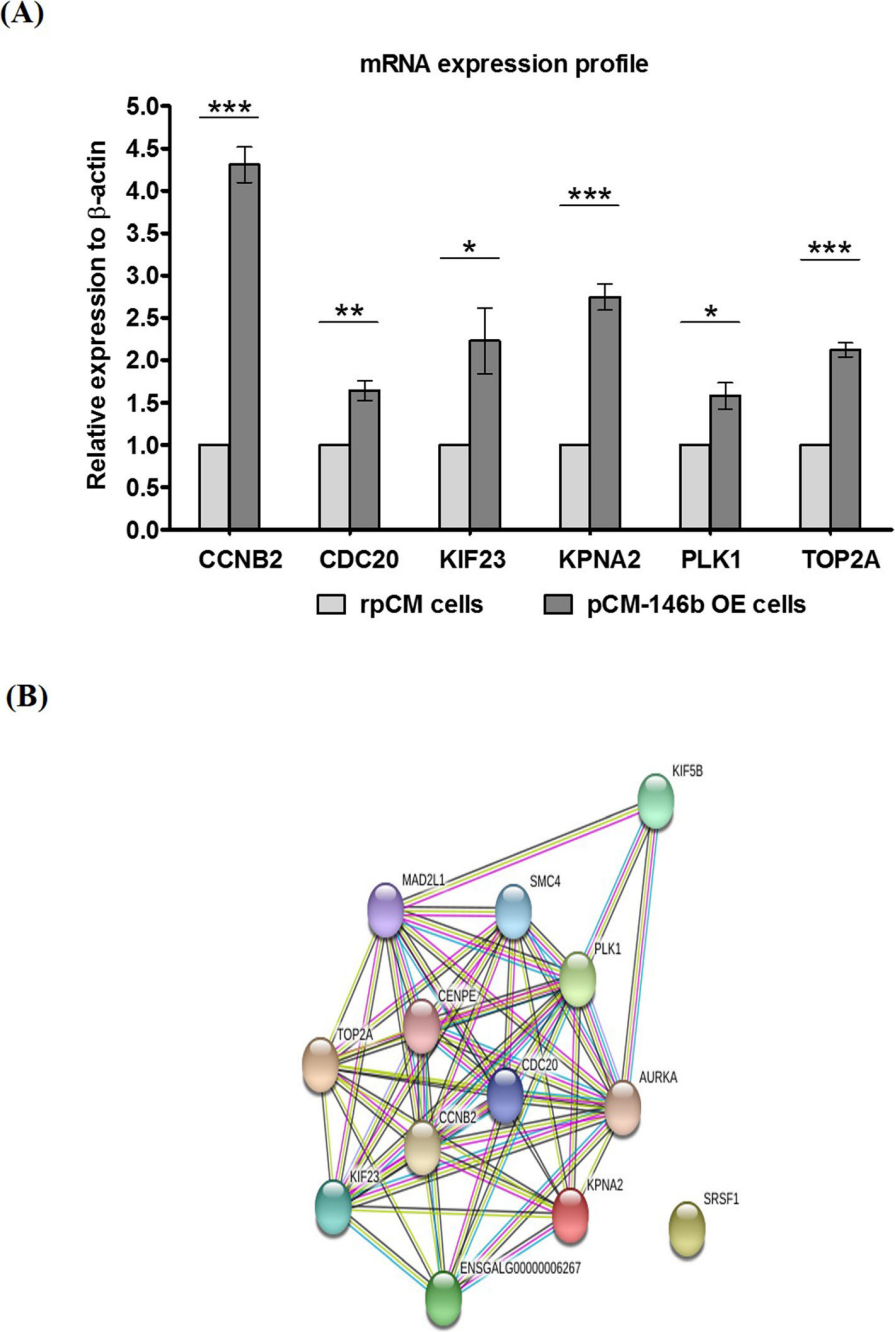
Validation of mRNA sequencing data and string analyses. **a** Expression profiles for upregulated genes (*Ccnb2*, *Cdc20*, *Kif23*, *Kpna2*, *Plk1*, and *Top2a*) in primary chicken myoblast (pCM) cells overexpressing miRNA-146b-5p (pCM-146b OE cells) by qRT-PCR (*n* = 3; ***p < 0.001). **b** String analyses of upregulated gene sets in the pCM-146b OE cells

Next we conducted mRNA sequencing analyses to compare global gene expression patterns between rpCM and pCM-146b OE cells. From mRNA sequencing data, we sorted out a total of 647 DEGs, of which 291 were downregulated and 356 were upregulated (Fig. 4a). Using fold change cutoffs of ≥1.5 (*p* < 0.001) and ≤ 0.6 (*p* < 0.001) for upregulated and downregulated gene sets, respectively, we identified four upregulated and two downregulated gene sets in the gene set enrichment analyses (http://software.broadinstitute.org/gsea/index.jsp; Table 1). Subsequently, heatmap analyses for mRNA sequencing data were conducted to visualize the different expression patterns of the predicted target transcripts and genes related to the cell cycle analyzed in Figs. 2 and 3, respectively (Fig. 4c). The gene sets globally up- and downregulated in pCM and pCM-146b OE cells were also visualized (Fig. 4d). To validate the DEGs from mRNA sequencing analyses, we analyzed the gene expression patterns of six upregulated genes (*CCNB2*, *CDC20*, *KIF23*, *KPNA2*, *PLK1*, and *TOP2A*) related to cell cycle regulation. All six transcripts were highly upregulated in pCM-146b OE cells (Fig. 5a). To understand the functional interactions between the upregulated genes and their neighbor genes, we performed STRING analyses (Fig. 5b). The results indicated that miR-146b-5p affected skeletal muscle proliferation and also influenced the regulatory pathways of cell cycling in pCM cells.

**Table 1:** List of up- and down-regulated gene sets

**Up-regulated Gene Sets Name**	**Description**	**Count.**	***p*-value**
E2F targets	Genes encoding cell cycle related targets of E2F transcription factors.	24	6.09E-23
G2/M checkpoint	Genes involved in the G2/M checkpoint, as in progression through the cell division cycle.	24	6.09E-23
Mitotic spindle	Genes important for mitotic spindle assembly.	13	1.23E-09
MYC targets V1	A subgroup of genes regulated by MYC – version1 (V1).	13	1.23E-09
**Down-regulated Gene Sets Name**	**Description**	**Count.**	***p*-value**
Xenobiotic metabolism	Genes encoding proteins involved in processing of drugs and other xenobiotics.	5	8.56E-04
Fatty acid metabolism	Genes encoding proteins involved in metabolism of fatty acids.	4	2.72E-03

### Overexpression of miR-146b-5p influences myotube differentiation in pCM cells

**Fig. 6:**
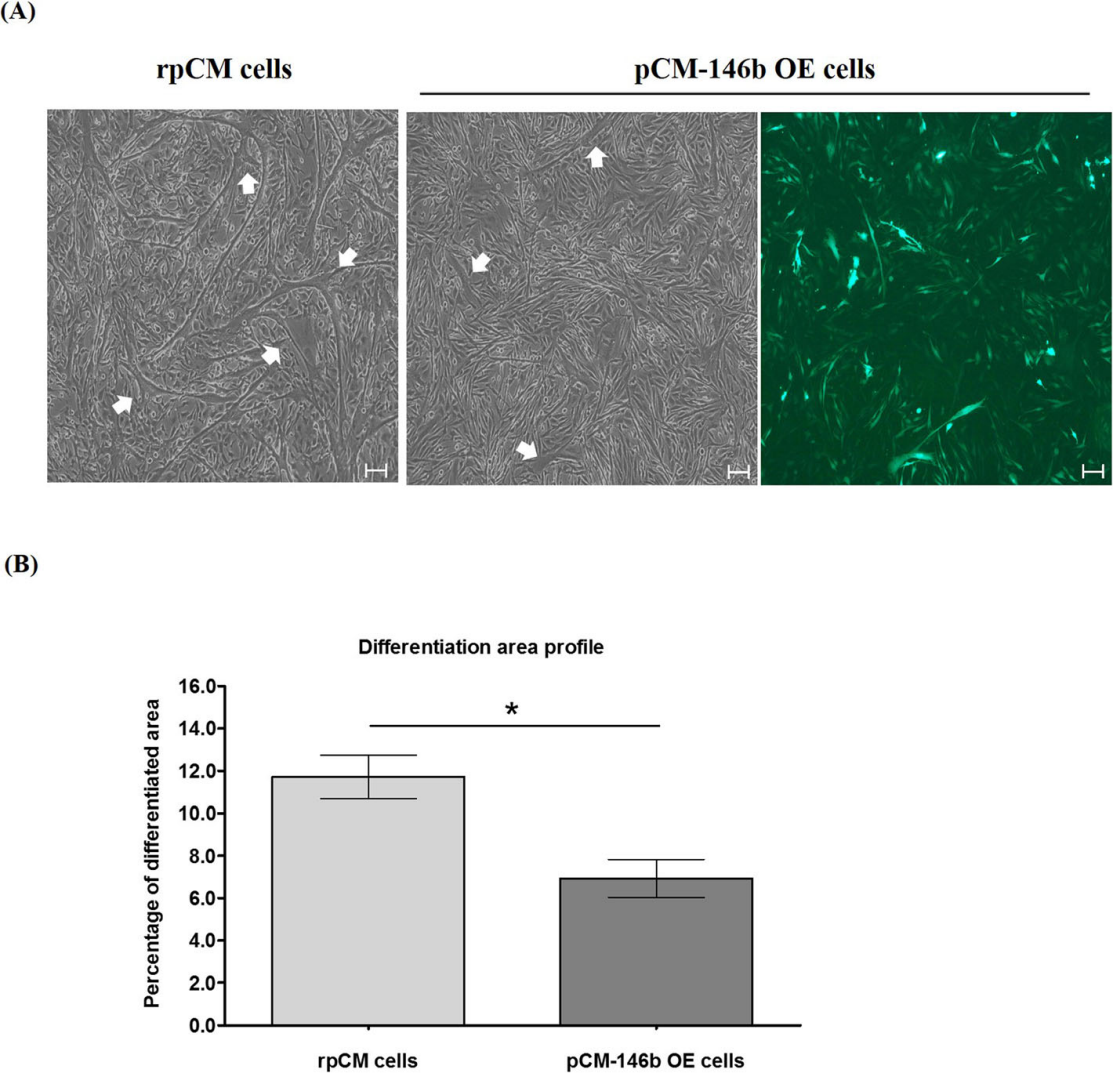
Morphological analyses of differentiated pCM cells overexpressing miR-146b (pCM-146b OE). **a** Morphological comparison of differentiated myotubes of regular pCM (rpCM) and pCM-146b OE cells after 4 days of differentiation. GFP was constantly expressed after myotube differentiation, which suggests that miR146b-5p was also expressed during and after differentiation. White arrows show the differentiated area (scale bar = 100 μm). **b** Comparison of the percentage of the differentiated area in rpCM and pCM-146b OE cells (*n* = 3; *p < 0.05)

**Fig. 7:**
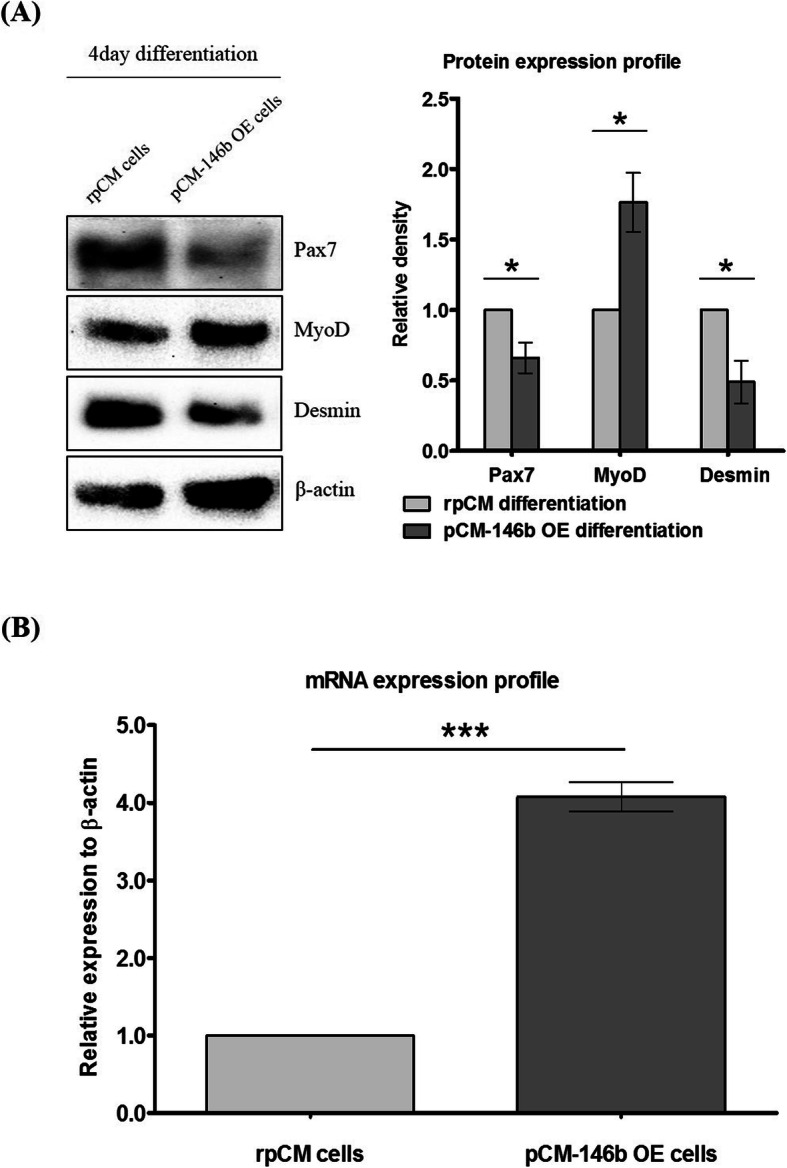
Protein expression analyses during differentiation and ID1 expression analyses. **a** Protein expression profiles of PAX7, MYOD, and Desmin were compared between regular pCM (rpCM) cells and pCM cells overexpressing miR-146b (pCM-146b OE) after 4 days of myotube differentiation. The graph represents a density comparison of Western blotting results (*n* = 3; *p < 0.05). **b** Expression profiles of ID1 were compared between the rpCM cells and pCM-146b OE cells (*n* = 3; ***p < 0.001)

We compared and analyzed myotube differentiation capacity between rpCM and pCM-146b OE cells. Overexpression of miR-146b-5p dramatically reduced myotube differentiation and formation during myogenesis in pCM-146b OE cells (Fig. 6). pCM-146b OE cells showed fewer differentiated myotubes and less myotube differentiation compared to rpCM cells (Fig. 6a). The area of differentiated myotubes was significantly decreased in pCM-146b OE cells after 4 days of myogenic induction (Fig. 6b). Western blotting showed similar expression patterns in cells in the undifferentiated stage and after the myogenic differentiation (Fig. 7a). Expression of PAX7 was still downregulated, whereas expression of MYOD was upregulated in pCM-146b OE cells. Furthermore, Desmin, a myogenic marker of terminal differentiation [4, 5], was also downregulated in pCM-146b OE cells. In addition, we investigated expression of ID1, which is closely associated with muscle differentiation by binding E proteins (Fig. 7b). Expression of ID1 was significantly upregulated in pCM-146b OE cells. These results demonstrate that overexpression of miR-146b-5p affects the expression of genes associated with myogenic differentiation and support the phenotypic difference between rpCM and pCM-146b OE cells after myogenic differentiation.

## Discussion

In this study, we showed that miR-146b-5p regulates myogenic proliferation and differentiation in pCM cells. A previous report suggested that chicken miR-146b-3p regulates myoblast proliferation and differentiation by negatively controlling the PI3K/AKT1 pathway and MDFIC [21]. In addition, mouse miR-146b-5p promotes myogenic differentiation in muscle cells by regulating computationally predicted target genes [20]. Chicken miR-146b-3p and mouse miR-146b-5p show different seed sequences and target genes, which indicates that the functional activity of miRNAs in the previous reports could be different from that of chicken miRNA-146b-5p in chicken muscle cells. To date, there are no data on the influences of chicken miR-146b-5p on the growth and differentiation of myoblast cells. Therefore, we investigated the effects of miR-146b-5p overexpression during myogenic differentiation in pCM cells. In a previous study, the *piggyBac* transposon was a stable system that integrated the miRNA expression transgene into the chicken genome [22–24]. Thus, we used the *piggyBac* transposon vector to overexpress miR-146b-5p and to deliver genes into the cells. miR-146 OE pCM cells had similar morphological features to rpCM cells but a faster growth rate.

pCM-146b OE cells showed higher expression of genes related to cell proliferation (*Ccnd3*, *Irf2*, *Wnt5a*, and *Pdgfrb*). The muscle of adult *Ccnd3*-null mice shows decreased proliferation and impaired G1/S progression, which suggests that CCND3 may be critical for muscle proliferation and cell cycle progression [25]. IRF2 indirectly improves muscle proliferation and regeneration by binding to the *VCAM-1* gene promoter in C2C12 cells [26]. Overexpression of WNT5A significantly increases the proliferation of satellite cells derived from mouse muscle and enhances cell division by promoting the nuclear translocation of activated β-catenin [27]. PDGF-induced effects have been observed in C2C12 skeletal muscle cells, resulting in stimulated skeletal muscle proliferation and inhibited myogenic differentiation [28]. Thus, pCM-146b OE cells show that the higher expression of PDGFRB mediated by miR-146b-5p might enhance cell proliferation and inhibit myogenic differentiation. We conducted RNA sequencing in rpCM and pCM-146b OE cells. Based on the RNA sequencing data, we identified upregulated gene sets that were closely related to the cell cycle (Table 1). The transcriptional levels of gene sets related to the cell cycle that were upregulated in pCM-146b OE cells demonstrate that they were reciprocally associated. miR-146b-5p activates and stimulates the regulatory circuits of the myogenic cell cycle and proliferation in pCM cells.

By contrast, myogenic differentiation is suppressed because myotube formation can be induced generally when the cell cycle is arrested [29]. To analyze the functionality of miR-146b-5p during myogenic differentiation, we examined myotube formation patterns and related gene expression after 4 days of differentiation. Although expression of MyoD was upregulated in the differentiated stage and in the undifferentiated stage, pCM-146b OE cells showed a lower differentiation capacity than rpCM cells after 4 days of differentiation (Fig. 6). Similarly, expression of Desmin, a myotube terminal differentiation marker [4, 5], was downregulated in pCM-146b OE cells. Moreover, expression of ID1 protein, an inhibitor of myogenic differentiation in muscle, was upregulated in pCM-146b OE cells (Fig. 7). ID1 protein competitively suppresses the E protein/MYOD complex because it has a higher affinity for the E-protein than MYOD and therefore inhibits myogenic differentiation [30, 31]. mRNA and protein levels of MYOD expression were upregulated, but myotube differentiation was significantly decreased in pCM-146b OE cells (Figs. 6 and 7). It is possible that the upregulation of ID1 protein controlled by miR-146b-5p blocked the E protein/MYOD complex. Furthermore, according to the integrated miRNA annotation and deep-sequencing database (http://www.mirbase.org), *SIRT1*, a target of miR-146b-5p, helps FOXO transcription factors to bind their target by deacetylation [32, 33]. FOXO3 transcriptionally represses ID1 by directly binding its promoter [34]. This suggests that miR-146b-5p can indirectly control the expression of ID1 protein by inhibiting SIRT1/FOXO3 and the regulatory pathway of myogenic differentiation by regulating the expression of ID1 protein.

## Conclusion

In this study, we generated pCM-146b OE cells and conducted a functional assay during myogenic proliferation and differentiation. Compared to rpCM cells, pCM-146b OE cells demonstrated higher proliferation and lower differentiation. pCM-146b OE cells had higher expression of genes related to cell proliferation and the cell cycle. The increased myogenic proliferation suggests that miR-146b-5p can enhance cell proliferation and inhibit myogenic differentiation by regulating the expression of PDGFRB. Furthermore, pCM-146b OE cells demonstrated higher expression of ID1, which indicates that miR-146b-5p can indirectly control myogenic differentiation by regulating the expression of ID1. These results suggest that miR-146b-5p acts as a key regulator of myogenic proliferation and differentiation in chicken.

## Methods

### Animal care

The procedures for animal management, reproduction, and manipulation adhered to the standard operating protocols of our laboratory at the University Animal Farm, Pyeongchang campus, Seoul National University, South Korea [24].

### Primary chicken myoblast (pCM) cell culture and myotube differentiation assay

According to our previous report [24], pCM cells were isolated from the pectoralis major of 10-day-old male chick embryos and maintained in Medium 199 (Invitrogen, Carlsbad, CA, USA; cat#11150–059) supplemented with 10% fetal bovine serum (FBS; HyClone Laboratories, Logan, UT, USA; cat#SH30084.03), 2% chicken serum (Sigma-Aldrich, St. Louis, MO, USA; cat#C5405), and 1× antibiotic-antimycotic (Invitrogen; cat#15240–062) [24]. These cells were cultured in an incubator at 37 °C in an atmosphere of 5% CO_2_ and 60–70% relative humidity. To differentiate into myotube at 80% confluency of cells, the cells were washed once in phosphate-buffered saline (PBS), and the differentiation medium containing 0.5% FBS and 1× antibiotic-antimycotic was changed. The differentiation medium was replaced with fresh differentiation medium daily according to our previous report [24]. The myotube-differentiated area was measured and analyzed in each well of regular pCM (rpCM) or pCM cells overexpressing miRNA-146b-5p (pCM-146b OE cells) after 4 days of differentiation. All experiments were performed in triplicate with both rpCM or pCM-146b OE cells.

### miR146b-5p overexpression vector construction

To overexpress chicken microRNA-146b-5p (miR-146b-5p), we inserted miR-146b-5p into the *piggyBac* transposon transgene expression system vector (System Biosciences, Palo Alto, CA, USA; cat#PB513B-1) after *Asc* I digestion and ligation (*piggyBac* cytomegalovirus [CMV]-GFP-miRNA-146b-5p). The CMV and elongation factor-1 promoters controlled the expression of GFP-miRNA-146b-5p and the puromycin resistance gene, respectively (Fig. 1a). The miRNA-146b-5p was synthesized as 5′-gct ggt gac gtc ccc tat gga att gag ttc tcc gct gtg aca ctt caa act gag aac tga att cca tag gcg atg tgg tca gca-3′ (Bionics, Seoul, Korea).

### Development of the miR-146b-5p overexpressing myoblast

For miR-146b-5p-expressing myoblast cells, we conducted co-transfection of the transgene expression vector, *piggyBac* CMV-GFP (control), or *piggyBac* CMV-GFP-miRNA-146b-5p with *piggyBac* transposase using Lipofectamine 3000 (Invitrogen; cat#L3000015) according to our previous report [22]. Transgene DNA-lipid complex consisting of 7.5 μL Lipofectamine 3000 reagent in 250 μL Opti-MEM (Invitrogen; cat#31985–062) and 10 μL P3000 reagent with 2.5 μg *piggyBac* transgene vector and *piggyBac* transposase plasmid in 250 μL Opti-MEM was added to each well according to our previous report [22]. One day after lipofection, 10 μg/mL puromycin was added to develop stably transfected cells with the transgene. GFP-expressing cells were observed with a fluorescent microscope (Carl Zeiss Axio Observer A1, Oberkochen, Germany).

### Analysis of gene expression by quantitative RT-PCR

Total RNA was extracted with Trizol reagent (Invitrogen; cat#10296010) and cDNA was synthesized with 2 μg RNA and random primers (Invitrogen; cat#18080–051) under standard conditions. Quantitative RT-PCR (qRT-PCR) for miRNA was conducted with the high-specificity miRNA QPCR Core Reagent Kit (Agilent Technology, Santa Clara, CA, USA; cat#600545). Each 20 μL RT-PCR reaction mix contained 2 μL cDNA, 2.5 μL PCR buffer, 1 μL dNTP mixture (2.5 mM), 1 U Taq DNA polymerase, and 10 pmol forward and reverse primers (Table 2). Quantitative RT-PCR analyses were performed with the iCycler iQ Real-time PCR detection system (Bio-Rad, Hercules, CA, USA) and EvaGreen (Biotium, Fremont, CA, USA; cat#31000). The PCR parameters were as follows: an initial incubation at 94 °C for 5 min, followed by 40 cycles at each condition (Table 2). The reaction was terminated by a final incubation at 72 °C for 10 min, and melting curve profiles were analyzed.

**Table 2:** List of primer sets for PCR analysis

**Gene**	**Forward**	**Reverse**	**Annealing Temp. (**°C**)**	**PCR size(bp)**
*Actin*	GATGATATTGCTGCGCTCGT	GTGCTCCTCAGGGGCTACTC	60 °C	618
*Pax7*	AGGTACCAAGAGACGGGCTC	CTCGGCAGTGAAAGTGGTCC	60 °C	411
*MyoD*	ACACGTCGGACATGCACTTC	TCTGACTCCCCGCTGTAGTG	55 °C	433
*SMAD4*	GCCCACCACAACATACTCCT	GCACTTGAGATCGAAGGCGT	60 °C	315
*NUMB*	GCTGCCCCAACTACTACTGC	ACAGGGCACTAATGCTGTCC	55 °C	310
*ADCK3*	CTGTGCAGCAAACATGTCCT	GGCATCTTCCATTTCCTTGA	60 °C	366
*RRM2B*	GGACCTTCCTCACTGGAACA	TCCACTTCAGAGCCCAGTCT	55 °C	308
*SGCB*	CACGAGTTTCATCTGCCAAA	TCACTTGCACCTTGAACAGC	55 °C	343
*CCND3*	TTTCTGGATGCTGGAGGTGT	ATGCAGAGCTTCTCCACAGT	60 °C	195
*IRF2*	AATGCAGAGGGACGACTTCA	ACTGGGTGATGTCTGACGTT	60 °C	301
*WNT5A*	GATACCGCTTTGCCAAGGAG	GCCTACCTTGCGGAAATCAG	60 °C	224
*PDGFRB*	AGAGCTAGAGGACAGTGGGA	CATTGGAAGCTCGGATGGTG	60 °C	359
*CCNB2*	TGAAATGTTGGTGGTAGGGC	GGAACAAGTATGCAAGTAGC	60 °C	209
*CDC20*	GAGTCCTGAACCTGACCATG	CTGTACAGTGTGTAAGCCCA	60 °C	221
*KIF23*	CCTTTCTTGTCAGGCCCTCT	TCTGTGAGCACGTTACCCTT	60 °C	348
*KPNA2*	ACACAGAGCAAGGGGTTACA	TCCAAATTCAGGGCAATGCT	60 °C	332
*PLK1*	CTGATGCTGTGGTGATGGTG	TCTCAACCTGGGCACGTTAA	60 °C	316
*TOP2A*	TCAACAAAGGCAGCAAGGTC	GGCTCGATTCATCCTGGAGA	60 °C	348
*ID1*	TGATCGACTACATCTGGGACC	TCTGAGAAGGTTACGAGCCG	60 °C	251
sno RNA: GGGATGTAAAAAAATACTTGCTATC miR-146b: UGAGAACUGAAUUCCAUAGGCG			60 °C	

### Western blotting assay

After extraction with 1× radioimmunoprecipitation lysis buffer, total protein from each treated cells was separated on a 10% polyacrylamide gel and transferred to a nitrocellulose membrane (Bio-Rad). The primary antibodies used were mouse anti-β-actin (Santa Cruz Biotechnology, Dallas, TX, USA; cat#SC-47778), anti-PAX7 (R&D Systems, Minneapolis, MN, USA; cat#MAB1675), anti-MYOD (Santa Cruz Biotechnology; cat#MAB1675), anti-Desmin (Novus Biologicals, Littleton, CO, USA; cat#NB110–1790). Horseradish peroxidase-conjugated anti-mouse IgG or anti-rabbit IgG (Bio-Rad; cat#170–6516 and 170–6515) were used as secondary antibodies. The blots were treated with ECL substrate solutions and exposed in a ChemiDoc XRS System (Bio-Rad) to detect chemiluminescence according to our previous report [24].

### Cell growth analyses

For analysis of the cell growth, we subcultured pCM-GFP or pCM-146b OE cells in 24-well culture plates (2 × 10^4^ cells/well). The total number of cells in each well was counted and analyzed statistically during a 5-day in vitro culture. In addition, the proliferative capacities were compared with a 5-bromo-2′-deoxyuridine (BrdU) flow kit (Becton, Dickinson and Company, Franklin Lakes, NJ, USA; cat#559619). Briefly, flow cytometry analyses of cell cycles were compared between rpCM and pCM-146b OE cells after the incorporation of BrdU.

### RNA-sequencing and data analyses

To analyze RNA-sequence data in rpCM cells or pCM-146b OE cells, we generated an RNA-sequencing library for each sample and carried out using Illumina HiSeq2500 (Illumina, San Diego, CA). Subsequently, these sequences were aligned and mapped against the chicken reference genome using TopHat for paired-end sequences according to our previous report [7]. Based on searches of the DAVID (http://david.abcc.ncifcrf.gov) and Medline (http://www.ncbi.nlm.nih.gov) databases, we conducted gene classification analysis. We identified differentially expressed genes (DEGs) from rpCM cells and pCM-146b OE cells with a *p* cutoff of 0.001 and a fold change cutoff of 1.5. Protein–protein association was analyzed with STRING analyses to identify all functional interactions of DEGs (https://string-db.org).

### Statistical analyses

We conducted statistical analyses with SAS version 9.4 (SAS Institute, Cary, NC, USA) and the significance of differences was analyzed with a general linear model procedure.

## Supplementary information

**Additional file 1.** Western blots in Fig. 2 (C). The original Western blot images of Pax7, MyoD, and β-actin in the undifferentiated stages.

**Additional file 2.** Western blots in Fig. 7 (A). The original Western blot images of Pax7, MyoD, Desmin, and β-actin after myotube differentiation.

## Data Availability

The datasets used during the current study are available from the corresponding author on reasonable request.

## Abbreviations

miRNA: microRNA
miR-146b-5p: microRNA-146b-5p
pCM: Primary chicken myoblast
rpCM: Regular pCM
pCM-146b OE: miR-146b-5p overexpressing pCM
NGS: Next generation sequencing
MRFs: Myogenic regulatory factors
GFP: Green fluorescent protein
CMV: Cytomegalovirus
qRT-PCR: Quantitative RT-PCR
Pax7: Paired box protein 7
MyoD: Myoblast determination protein 1
SMAD4: SMAD family member 4
ADCK3: aarF domain containing kinase 3
RRM2B: Ribonucleotide-diphosphate reductase subunit M2 B
SGCB: Sarcoglycan Beta
CCND3: Cyclin D 3
IRF2: Interferon regulatory factor 2
PDGFRB: Platelet-derived growth factor receptor beta
DEGs: Differentially expressed genes
CCNB2: Cyclin B 2
CDC20: Cell-division cycle 20
KIF23: Kinesin-6
KPNA2: Karyopherin alpha 2
PLK1: Polo-like kinase 1
TOP2A: Topoisomerase 2-alpha
ID1: DNA-binding inhibitor 1
SIRT1: Sirtuin 1
FOXO3: Forkhead box O 3
