# Supplementary figures and images for: Functional analyses of miRNA-146b-5p during myogenic proliferation and differentiation in chicken myoblasts
Source: BMC Mol Cell Biol. 2020 May 29;21:40. doi: 10.1186/s12860-020-00284-z (PMC7260857; doi:10.1186/s12860-020-00284-z)

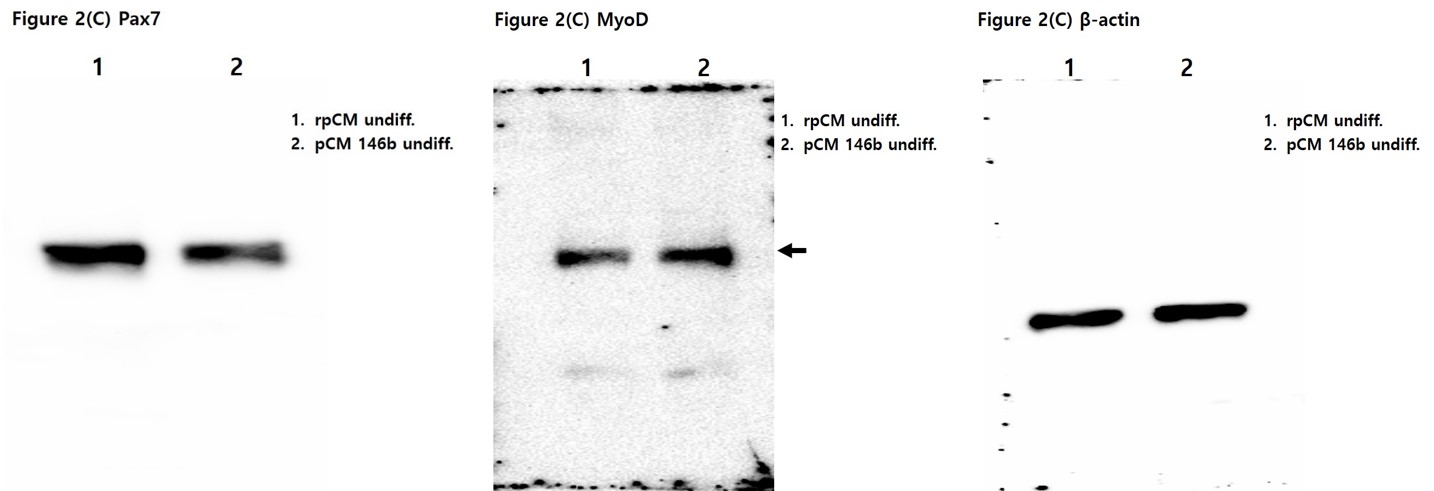

Supplement: Supplementary file 1 — Additional file 1. Western blots in Fig. 2 (C). The original Western blot images of Pax7, MyoD, and β-actin in the undifferentiated stages. [file 12860_2020_284_MOESM1_ESM.jpg]

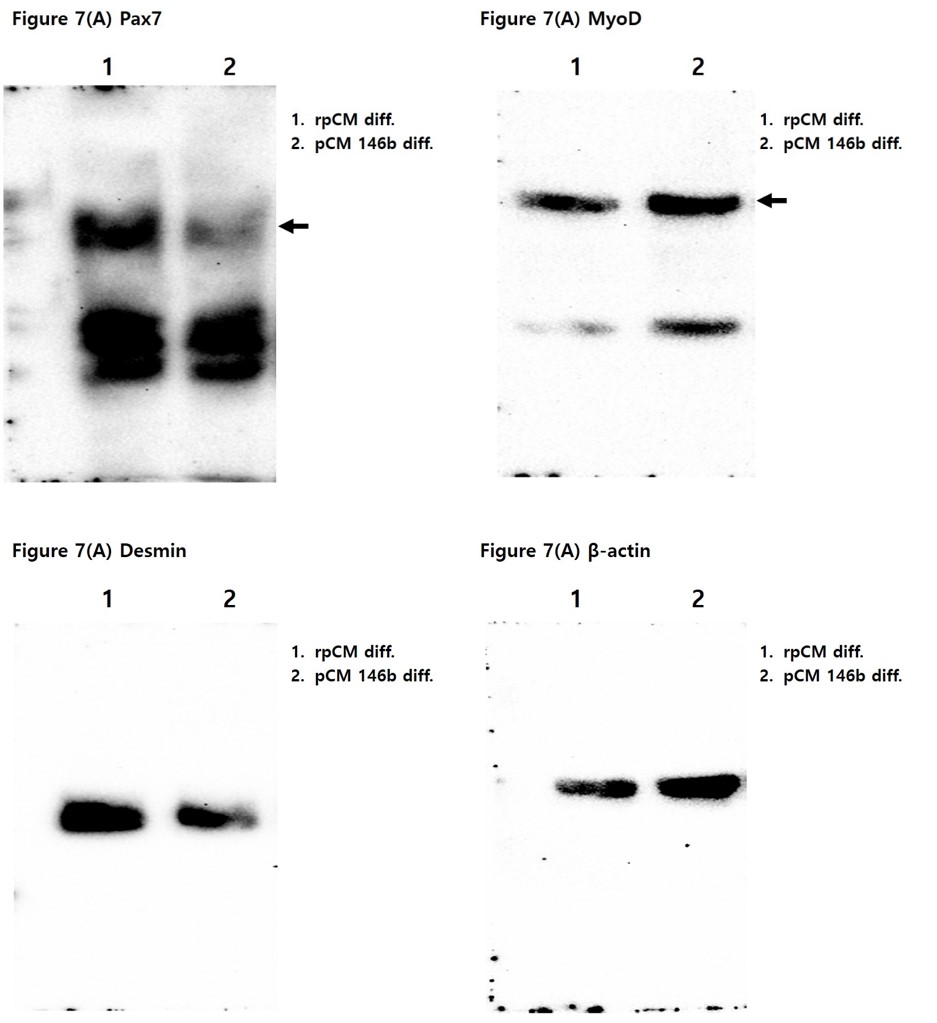

Supplement: Supplementary file 2 — Additional file 2. Western blots in Fig. 7 (A). The original Western blot images of Pax7, MyoD, Desmin, and β-actin after myotube differentiation. [file 12860_2020_284_MOESM2_ESM.jpg]
